# Recurrent spontaneous coronary artery dissection with ST-segment elevation: once RCA involvement then left main/LAD—case report

**DOI:** 10.1186/s43044-024-00510-5

**Published:** 2024-07-19

**Authors:** Sara Khaki, Vafa Baradaran Rahimi, Mostafa Ahmadi

**Affiliations:** Department of Cardiovascular Diseases, Faculty of Medicine, Mashhad University of Medical Sciences, Mashhad, Iran

**Keywords:** Left main coronary artery, Percutaneous coronary intervention, Spontaneous coronary artery dissection, Right coronary artery dissection, Acute coronary syndrome

## Abstract

**Background:**

Spontaneous coronary artery dissection is a rare disease with a more prevalence in women, mostly in the postpartum state, which was first described by Peretti in 1931.

**Case presentation:**

This report describes a previously healthy woman who had a spontaneous coronary artery dissection. This case is related to the early postpartum period with a successful outcome. In addition, the diagnostic and therapeutic approaches of this unique clinical entity are discussed and reviewed.

**Conclusions:**

Because these kinds of cases are so rare, reporting these cases and the management and treatment approaches can guide other clinicians worldwide, and maybe a guideline for choosing the best approach around different situations could be published.

## Learning points

This case report is important because it presents a novel and rare case of SCAD; also, the important management of this case and situation should be discussed all around the world.

## Background

Spontaneous coronary artery dissection (SCAD) involves the separation of the coronary artery wall layers without iatrogenic or traumatic intervention. It is possible to form a false lumen between the tunica intima and media or between the tunica media and the tunica externa [1]. It is the blood that flows into the tear. Sometimes, the blood stops and forms a thrombus at the site of the tear. Obstruction of the true lumen can occur due to increased pressure in the false lumen. Flow in the true lumen is reduced, leading to myocardial ischemia or infarction. It was recorded in 1931 that the first case of SCAD had occurred [2].

The severity of SCAD symptoms is usually correlated with the degree of obstruction. There are several symptoms that can occur, ranging from asymptomatic angina to ST-elevation acute myocardial infarction and possibly sudden death from heart arrhythmias. [3, 4]. The prevalence of SCAD in the catheterization laboratory population is 0.2%. About 0.07% of SCAD is found in men and 0.6% in women [5]. SCAD is especially more likely in young women with a history of cardiac or fibromuscular dysplasia (FMD). In females under 50 years of age with unstable angina or ST-segment elevation, SCAD is prevalent in 8.7% and 10.8%, respectively [6]. The most common coronary artery involved in SCAD is the left anterior descending (LAD), followed by the RCA. This case report is critical because it presents a new and rare case of SCAD occurring in the LAD and RCA. Also, the important management of this case and situation should be discussed worldwide.

## Case presentation

**Fig. 1:**
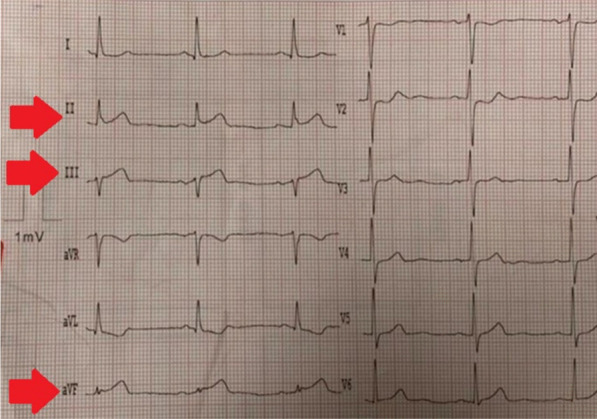
Red arrows show ST-segment elevation in inferior leads (Inferior STEMI)

**Fig. 2:**
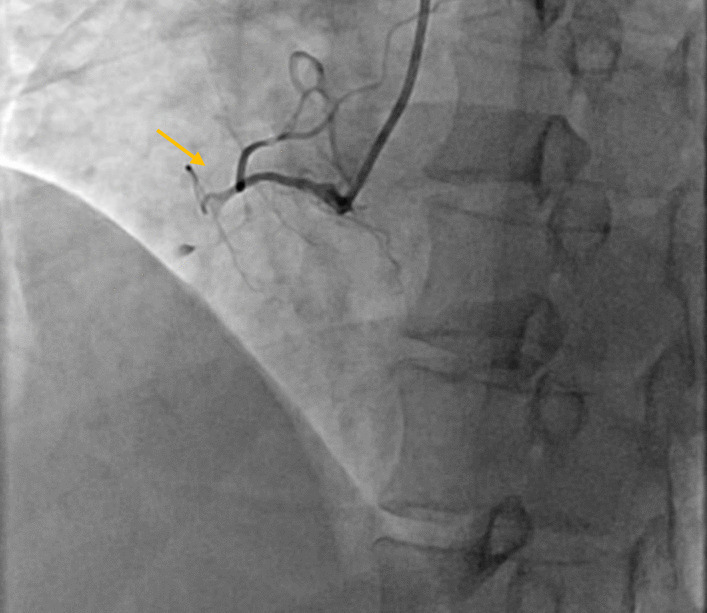
Occluded right-coronary–artery (yellow arrow)

**Fig. 3:**
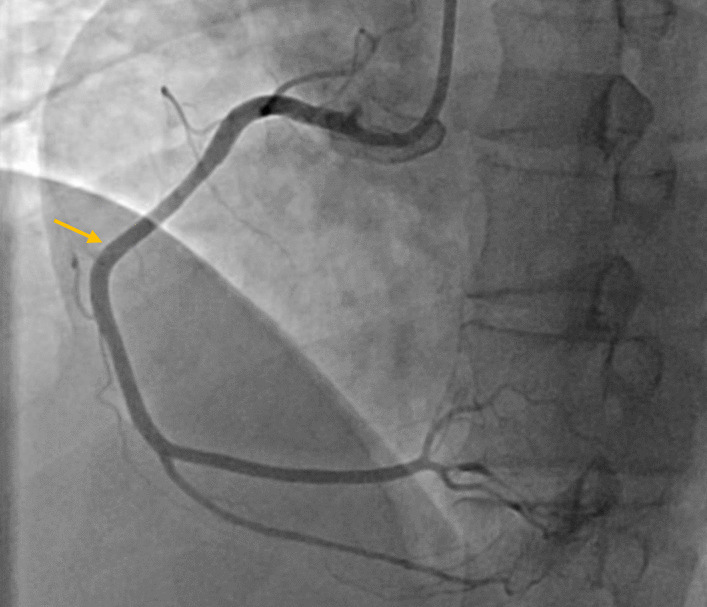
Right coronary artery with good TIMI flow after POBA and flap of dissection (yellow arrow)

A 40-year-old female in the postpartum stage was admitted to the emergency department with typical and intensive chest pain. On presentation, the clinical examination was normal; blood pressure and heart rate were 116/60 mmHg and 80 beats/min, respectively. Electrocardiography showed ST-segment elevation in inferior leads (Fig. 1). Transthoracic echocardiography showed akinesia of the mid to apex inferolateral, inferior, and inferoseptal walls, with an ejection fraction of 40–45%. Immediately, the patient was transferred to the interventional catheterization room (CATH LAB) and then underwent coronary angiography via the access of the right radial artery, which showed an occlusion at the ostioproximal part of the right coronary artery (Type 4 SCAD) (Fig. 2). After that, she went under percutaneous balloon angioplasty without stenting, and the result was an excellent TIMI flow (3/3) for the right coronary artery (illustrated in Fig. 3).

**Fig. 4:**
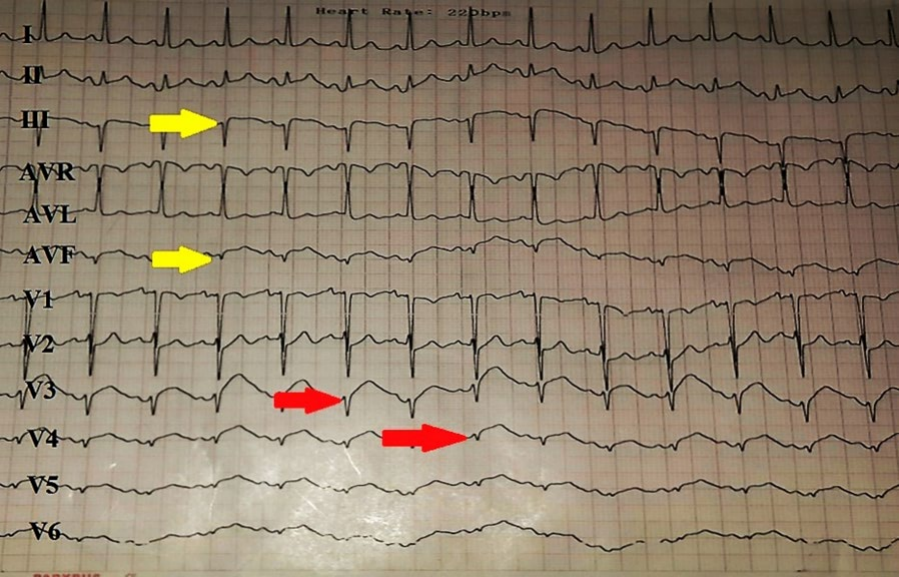
Red arrows show cover ST-segment elevation in precordial leads (anterior STEMI), and yellow arrows show pathological Q wave from previous inferior STEMI

**Fig. 5:**
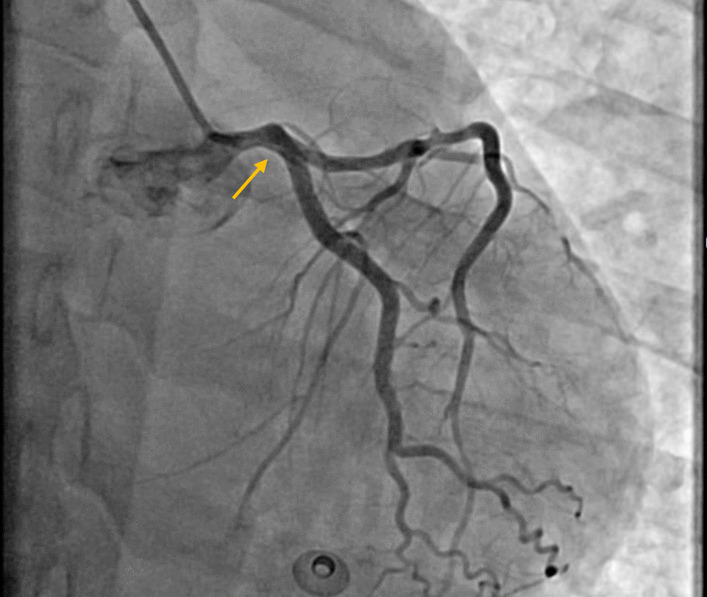
Type 1 SCAD at the proximal part of LAD with extending backward to LM (yellow arrow)

**Fig. 6:**
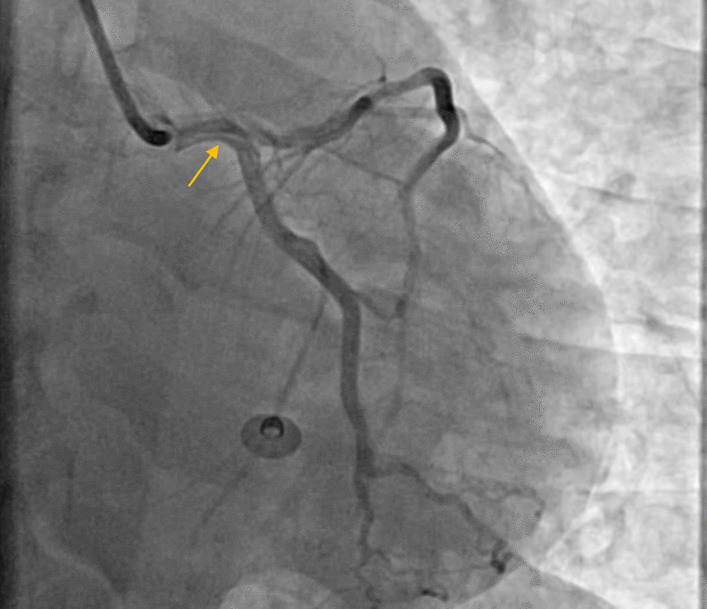
The flap of dissection of LM and LAD arteries (yellow arrow)

**Fig. 7:**
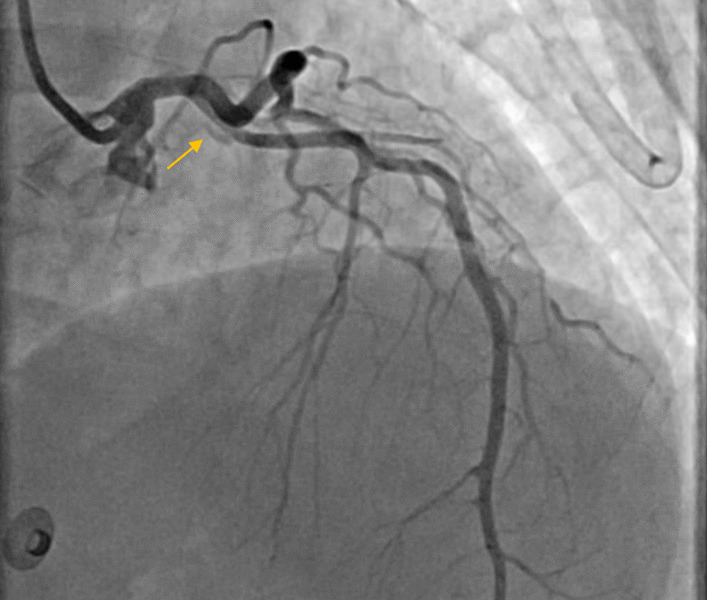
Flap of dissection with dye staining at the proximal part of LAD artery (yellow arrow)

Iliac and renal angiography were also done, and there was no evidence of arteriopathy. She was treated conservatively with aspirin, beta-blocker, and statin. She was admitted to CCU, and after three days of angiography and balloon angioplasty, she had typical chest pain again. The ECG revealed a convex appearance of ST-segment elevation in the anterior precordial leads associated with pathological Q waves (Fig. 4). The results of the transthoracic echocardiography showed hypokinesia of the apical and apicoseptal segments. The ejection fraction was 30–35%, so she was immediately transferred to CATH LAB, and coronary angiography via the radial artery showed a flap of spontaneous dissection of the LAD artery without any plaque or lesion (Figs. 5, 6 and 7).

The patient was treated conservatively with aspirin, bisoprolol, captopril, and a statin because LAD disease was thought to be SCAD. We evaluated her two months later with acceptable results.

## Discussion

SCAD is an infrequent etiology of ACS, mostly involving younger and healthier populations [7]. The clinical presentations have a lot of variety and can vary from only chest pain to ST-segment–stimulated myocardial infarction (STEMI), ventricular tachyarrhythmia, and sudden death. By the way, coronary artery involvement can be varied from single to multiple arteries [8].

A characteristic finding in SCAD is a dissection of the coronary intima or media, but also hematoma formation in the vessel wall often occurs. We do not know which is the primary event, dissection or hematoma, but both may result in ductal obstruction [9]. The best method to diagnose SCAD is invasive coronary angiography. However, other imaging techniques such as computed tomography angiography (CTA), intravascular ultrasound (IVUS), and optical coherence tomography (OCT) may help the physician to make a definite diagnosis [8].

If SCAD occurs during gestation or within 12 months after delivery, it is called pregnancy-associated SCAD (P-SCAD). It is estimated that about 5–10% of all SCAD cases are caused by P-SCAD [10]. In this setting, it is valuable to report that 10–22% of ACS events occur during pregnancy, and 23–67% of ACS events following childbirth were caused by P-SCAD. [10]. Several pieces of evidence support that P-SCAD is associated with greater involvement of proximal and distal dissections, which can lead to larger infarcts [11].

## Conclusion

The diagnosis of SCAD is important because there are substantial differences in the management of dissection-induced infarction compared with atherosclerotic ischemia. In most patients with SCAD, invasive coronary angiography provides a definitive diagnosis for physicians. There is insufficient evidence to add P2Y12 inhibitors to the treatment of nonvascular SCADs. The area of intima rupture is prothrombotic. Therefore, antiplatelet agents such as aspirin may theoretically reduce false lumen thrombosis. Long-term antiplatelet therapy with aspirin is the mainstay of treatment in our center. Statin administration is recommended for patients with underlying atherosclerotic CAD and preexisting dyslipidemia due to guideline indications.

## Data Availability

All data and materials pertaining to the index case are included in this published article.

## Abbreviations

CATH LAB: Catheterization room
CTA: Computed tomography angiography
FMD: Fibromuscular dysplasia
IVUS: Intravascular ultrasound
LAD: Left anterior descending
OCT: Optical coherence tomography
P-SCAD: Pregnancy-associated SCAD
SCAD: Spontaneous coronary artery dissection
STEMI: ST-segment–stimulated myocardial infarction
